# Why Not?

**Published:** 1867

**Authors:** 


"*Why Not?*"—The somewhat peculiar title of the useful monograph noticed on another page, would scarcely have been chosen by the author had he been a resident of Chicago. The classic shades, "Under the Willow," which but recently were lightened up by the sun of advancing civilization, for many years had this sign in gilded letters over the principal entrance: "Why Not—Roger Plant's Saloon."

